# Enhanced Air Filtration Efficiency through Electrospun PVC/PVP/MWCNTs Nanofibers: Design, Optimization, and Performance Evaluation

**DOI:** 10.1021/acsomega.4c03628

**Published:** 2024-08-24

**Authors:** Armando A. Escriba Flores, Daniela Sanches de Almeida, Monica Lopes Aguiar, Carlos Eduardo Cava

**Affiliations:** † Federal University of Technology − Paraná, Av. Dos Pioneiros, 3131, Londrina, PR 86036-370, Brazil; ‡ Federal University of São Carlos, Rod. Washington Luiz, km 235, SP310, São Carlos, SP 13565-905, Brazil

## Abstract

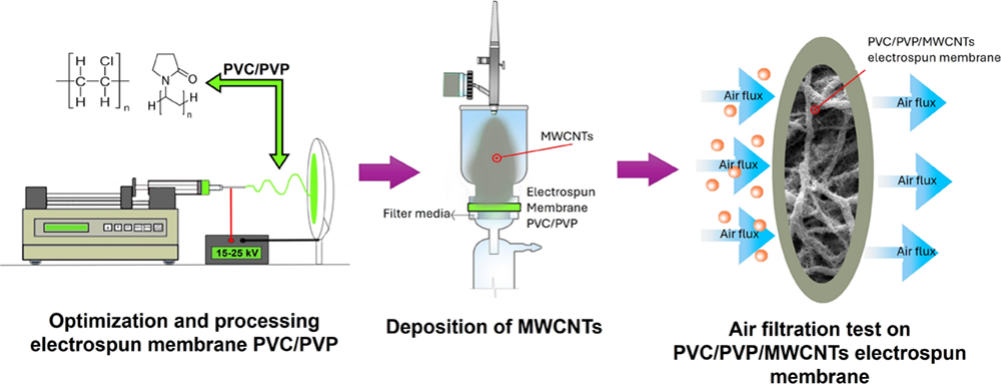

This study presents a novel approach for creating an effective air filtration medium using electrospun nanofibers comprised of poly(vinyl chloride) (PVC), poly(vinylpyrrolidone) (PVP), and impregnated with multiwall carbon nanotubes (MWCNTs). The membrane production was optimized using an experimental design methodology, resulting in a hydrophobic membrane that exhibits excellent dispersion of MWCNTs. Scanning electron microscopy images illustrate the nanofibers’ morphology, featuring an average diameter of approximately 240 nm, minimal bead formation, and optimal MWCNT dispersion. Air filtration tests conducted with NaCl nanoparticles (7–300 nm) demonstrated superior permeability (10^–12^ m^2^) and minimal pressure drop (approximately 780 Pa at a 5 LPM airflow rate) compared to other electrospun materials. Both MWCNT-impregnated samples and individual PVC/PVP nanofibers exhibited filtration efficiencies nearing 96%. These results underscore the potential of this developed material for air filtration, particularly in indoor environments, where MWCNTs effectively adsorb and maintain low levels of gaseous and particulate pollutants. This study emphasizes the design, optimization, and comprehensive performance evaluation of PVC/PVP/MWCNT nanofibers, showcasing significant advancements in filtration efficiency with high flux. The findings suggest promising applications for this composite material in advanced air purification systems.

## Introduction

Air pollution, driven by the rapid expansion of the global population, stands as a formidable challenge to both environmental sustainability and public health. The intricate composition of the atmosphere, laden with diverse pollutants such as aerosols, gases (e.g., carbon monoxide, ozone, nitrogen oxides, and sulfur dioxide), and potential airborne pathogens like SARS-CoV, underscores the urgent need for effective air filtration methods.^1−13^ Among these methods, air filtration plays a pivotal role as a longstanding separation technique crucial for mitigating pollution’s adverse effects by capturing harmful particles and pathogens, thereby enhancing indoor air quality.^14^

The efficacy of air filtration hinges significantly on physical barriers that impede particle flow while facilitating airflow, where the pore size and membrane structure emerge as primary considerations. Porous membranes and fibrous materials excel in retaining specific particle sizes and managing associated pressure drops, directly impacting energy consumption.^14^ However, filters that excel in capturing small particles often suffer from increased thickness, leading to increased pressure drops. Thus, efforts are crucial to optimizing efficiency while minimizing these drops.^15^ Fibrous materials offer a compelling advantage by addressing efficiency and pressure drop concerns simultaneously.^16,17^ Theoretical foundations elucidate key mechanisms driving particle retention in fibrous media: diffusion, interception, impaction, gravitational settling, and electrostatic attraction.^15,18,19^ This understanding forms the basis for designing tailored filtration configurations that account for particle size, flow dynamics, structural attributes, and functionalization, promising high-performance filtration with minimal pressure drop.^15^

Electrospinning has emerged as a promising technique for fabricating membranes, enabling the formation of nano or microfibers that construct structured, interconnected porous membranes.^20−24^ This process is initiated with the placement of a polymer solution in a syringe pump equipped with a needle. An electric current connects the system with one electrode at the needle tip and another on the substrate. The electrical potential between these electrodes generates a jet of fibers, shedding solvent during trajectory and depositing on the substrate, easily removable. Simultaneously, the syringe pump continuously supplied the solution from the syringe tip to the collector. The technology is particularly appealing due to the small fiber diameter, reaching the nanometric scale, offering high surface area, and allowing a variety of materials use.^25,26^ These features render electrospinning suitable for diverse applications, including tissue engineering, controlled drug delivery, and industrial processes like air filtration,^27,28^ and contaminant adsorption.^20,29−32^

Incorporating nanomaterials into the polymer solution profoundly alters the resulting fiber physicochemical and mechanical properties. Studies show that these nanomaterials enhance selectivity in adsorption processes and increase fiber strength and elasticity modulus. Materials like pristine or functionalized multiwalled carbon nanotubes (MWCNTs), graphene, TiO_2_ nanoparticles, and silver or gold nanoparticles have been widely studied in fields such as medicine, water treatment,^33,34^ photocatalysis,^35^ and tissue engineering. For instance, Xiong Ranhua et al.^36^ demonstrated the creation of high-performance nanocomposites by incorporating iron nanoparticles into a PCL matrix, producing nanofibers approximately 300 nm in diameter, capable of delivering macromolecules to cultured cells upon laser stimulus.

Furthermore, while embedding nanomaterials within fibers has shown innovative results, depositing nanoparticles onto fiber surfaces, though less discussed in the literature, offers an alternative that enhances selectivity and efficiency. This approach is particularly appealing in filtration processes as nanomaterials can directly contact the medium, supported by electrospun nanofiber stability, thereby increasing surface area and filtration selectivity.

Among electrospinning polymers, poly(vinyl chloride) (PVC) stands out for its cost-effectiveness and abundant availability from recycling sources. Electrospinning can yield PVC fibers with diameters ranging from 100 to 500 nm, exhibiting low defects and excellent morphology.^29,30^ However, PVC fibers often lack the mechanical properties essential for effective filtration.^37^ Incorporating polyvinylpyrrolidone (PVP) can enhance mechanical stability, enabling effective filtration.^38,39^

This study outlines three primary research objectives. First, employing an experimental model is employed to construct and characterize electrospun PVC/PVP fibers with small diameters and minimal defects. Second, optimizing MWCNTs deposition via vacuum-assisted spray ensures comprehensive fiber coverage. Lastly, evaluating this filtration system includes assessing MWCNTs’ impact on particle filtration efficiency, analyzing particles ranging from 5 to 300 nm at a flow rate of 5 LPM.

## Methodology

### Materials

The electrospun fibers were obtained by considering the following materials: PVC (poly(vinyl chloride)) *M*_w_ 80,000 Da, (purchased from Sigma-Aldrich, USA), PVP (poly(vinylpyrrolidone)) *M*_n_ 40,000 Da (purchased from Dinâmica Qumica Contemporânea, Brazil), and *N*,*N*-dimethylacetamide, (DMAC) 99% (purchased from Neon reagents analticos, Brazil) were used as the solvent. The carbon nanotubes that were dispersed on the PVC/PVP electrospun fibers were purchased from (purchased from US Research Nanomaterials, Inc., USA) with purity >90%, external diameter between 20 and 40 nm, and surface area of 110 m^2^/g.

### Experimental Design

The electrospun fibers were constructed by using an experimental design to obtain PVC/PVP electrospun fibers with small diameters and without morphological defects. The initial values for PVC blends of electrospun fibers were chosen from the literature.^38,39^ The experimental design considers three parameters (23) to be analyzed: the concentration of DMAC/PVC in weight ratios, the percentage of the total weight of PVP, and the voltage applied. The concentration DMAC/PVC and the rate of PVP wt % had three levels of analysis for DMAC/PVC, 85/15, 80/20, and 75/25 in weight ratios and 3, 5, and 7% of the total weight of PVP. Four voltages, 15 and 25 kV, were analyzed. The parameters investigated the levels, and the code for each experiment can be consulted in the Supporting Information Table S1.

### Electrospinning Process

The polymers PVC and PVP solubilizations were performed following the experimental design. All the experiments considered 2 mg of PVC as a fixed value, and the addition reached a simple linear relation. In the case of experiment number 2 from Table S1, 2 mg of PVC and 0.3 mg of PVP were added to 8 mg of DMAC, and the solution was kept in magnetic stirring for 4 h at 70 °C to get a homogeneous solution. The solution was collected with a syringe and placed in a syringe pump. The electrospinning arrangement considers a distance between the tip of the needle and the collector of 25 cm; the feed rate was 1.2 mL/h, and the voltage was kept fixed at 15 kV. This process was repeated for all the experiments.

### Characterization of Electrospun PVC/PVP Nanofibers

The fiber morphology was analyzed at the beginning with an optic microscope (BA210, Motic) with 1000× magnification to study the surface morphology and estimate the diameter of the fibers for the experiments cited in Table S1. The experiments with better performance in small diameters and less quantity of beads were analyzed under scanning electron microscopy (SEM), model FEI Quanta 200. A representative sample of 200 fibers was analyzed to determine the average diameter of the membrane, and qualitative analysis determined which fiber contained fewer morphological defects. The morphology and dispersion of MWCNTs were analyzed (SEM), and the functional chemical groups were studied by infrared spectroscopy. The surface nature of the electrospun membrane PVC/PVP was examined by measuring the water angle contact (WAC). This experiment was repeated five times to confirm the values. The chemical functional groups present in the electrospun fibers were detected by infrared spectroscopy.

### Nanocomposite Preparation

**Figure 1:**
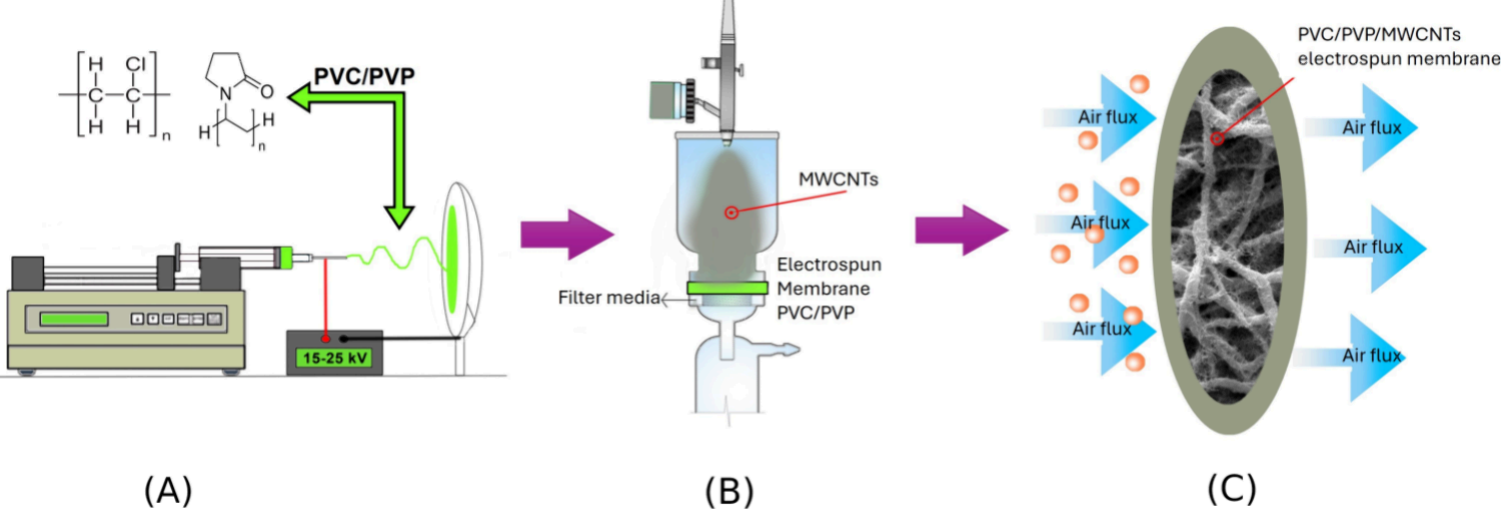
Optimized solution is used to obtain fibers with small diameters and few morphological defects (A); dispersion of nanotubes in water using an ultrasonic bath for proper deposition onto PVC/PVP nanofibers via the spray method (B); and evaluation of the membrane’s performance in air filtration (sky blue arrows indicate the air flow, and the orange balls represent the particles) (C).

The preparation of the composite first considered the dispersion of MWCNTs in water before deposition on the substrate (PVC/PVP electrospun nanofibers). Figure 1 shows the principal steps in the conformation of the nanocomposite. The MWCNTs dispersion consists of adding 0.9 mg of MWCNTs into 200 mL of deionized water and keeping it under an ultrasound bath for 3 h. A spray assisted by a vacuum pump was used for MWCNTs’ incorporation on the surface of the electrospun nanofibers. For spray deposition, the electrospun fiber membrane PVC/PVP was placed in a vacuum filter and 10 mL of the solution (MWCNTs/water) was deposited by spray.

### Air Filtration Test

The desirability tests found optimal conditions for producing PVC/PVP nanofibers. Thus, samples of nanofibers were made under optimal conditions to perform filtration performance tests. Hence, the produced membranes were framed in circular shape with 47 mm of diameter; the samples with carbon nanotubes (impregnated by spray) presented about 200 μm of thickness and those without had about 160 μm. The thermal stability of the samples was analyzed by thermogravimetry from 25 to 750 °C.

**Figure 2:**
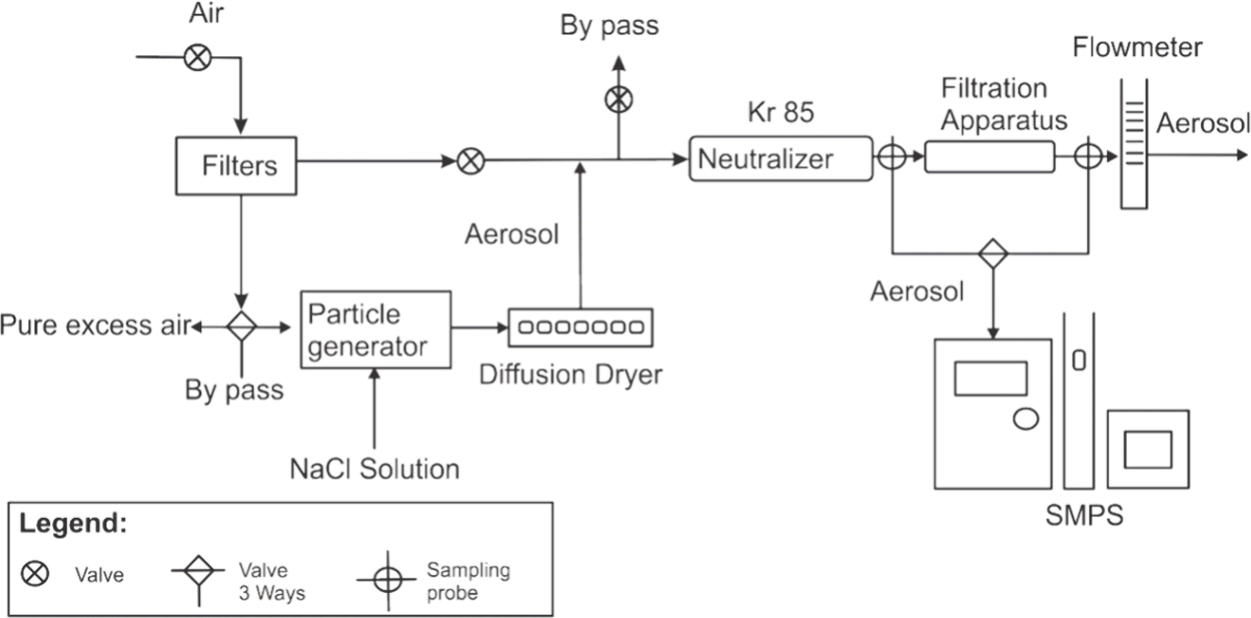
Schematic of equipment used for permeability and nanoparticle filtration efficiency tests.

The air filtration apparatus is composed of an air compressor (Shultz Shultz, Joinville, Brazil), air purification filters (Models A917A-8104N-000 and 0A0-000), aerosol generator (Model 3079, TSI, Shoreview-MN, USA), diffusion dryer (Norgren, Birmingham, UK), Krypton neutralization source (TSI Model 3054), filter holder, and flow meter (Gilmont Instruments, Inc., Barrington-IL, USA). In addition, the electrostatic classifier (TSI Model 3080), SMPS (Scanning Mobility Particle Sizer) differential mobility analyzer, and ultrafine particle counter (TSI Model 3776) were used to measure the nanoparticles before and after the filter,^4,5,40,41^ as shown in Figure 2.

For the permeability tests, the membranes were placed in the filtration apparatus through which pure air passes; the surface area of the filter in this system was 5.3 cm^2^. Thus, the pressure drop was measured in different flow rates (from 1 to 9 LPM); using Darcy’s law, it was possible to calculate the permeability constant (*k*1). The same apparatus was used for the filtration efficiency, with face velocity and airflow rate constant (16 cm/s and 5 LPM, respectively). These values were defined in our previous study, considering the behavior of nanofibers during permeability tests. Regarding face masks application, it was decided to set the air flow rate at 5 LPM to ensure that there would be no rupture of the nanofiber during the filtration efficiency test.^4^ Sodium chloride (NaCl) solution (0.1 g/L) generated nanoparticles for 15 min for each measure downstream and upstream of the nanofiber’s membrane. Then, the particle number and size distribution were measured to obtain the membrane efficiency in collecting nanoparticles.^40−42^

## Results and Discussion

Many parameters influence polymeric nanofiber production using the electrospinning technique, including rheological polymer characteristics, flux, applied voltage, distance from the electrode, and ambient conditions.^16^ Among these parameters, the ratio of DMAC/PVC is particularly crucial in forming electrospun fibers. This study analyzed three levels of this parameter: 85/15, 80/20, and 75/25. Optical analysis revealed that configuration 75/25 is unsuitable for forming electrospun fibers due to the accumulation of dry material in the needle, likely caused by rapid solvent evaporation. Configuration 85/15 (DMAC/PVC) showed potential for forming electrospun fibers, but a high quantity of beads was observed in all configurations for this ratio. This phenomenon is attributed to poor interaction with the polymer and the electric field, leading to instability in Taylor cone formation and morphological defects.

**Figure 3:**
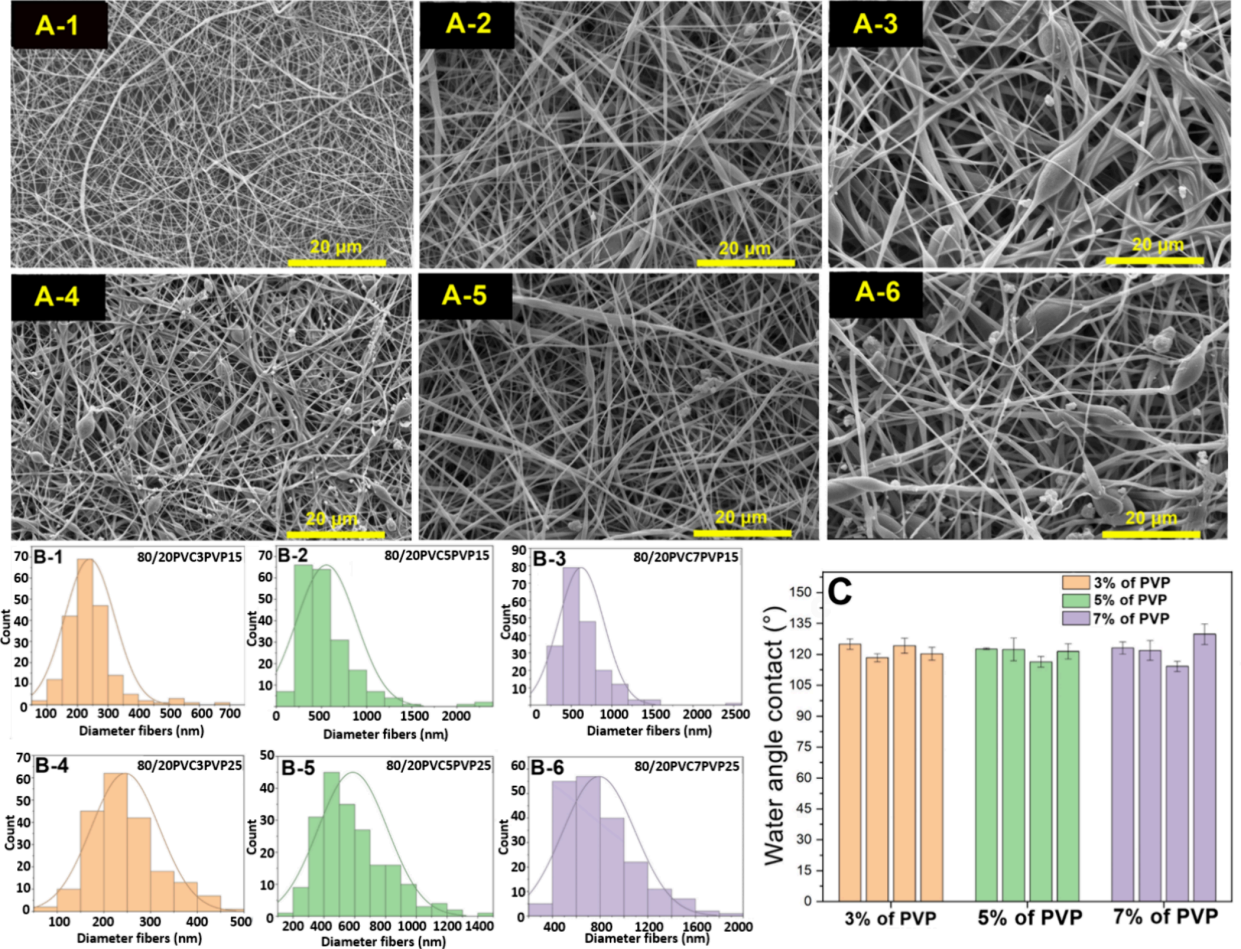
SEM images (A) and diameter distribution (B) of nanofibers obtained (1) 80/20pvc3pvp15, (2) 80/20pvc5pvp15, (3) 80/20pvc7pvp15, (4) 80/20pvc3pvp25, (5) 80/20pvc5pvp25, and (6) 80/20pvc7pvp25 and contact angle measurement behavior of all the experiments, which allows the formation of electrospun fibers (C).

In contrast, configuration 80/20 (DMAC/PVC) demonstrated improved results in forming electrospun fibers with smaller diameters and fewer morphological defects. Based on preliminary analysis after completing 18 experiments, samples with the ratio 80/20 (DMAC/PVP) were selected, reducing the total number of samples analyzed to six. SEM images in Figure 3A illustrate differences related to the concentration of PVP and applied voltage. Diameter and morphological defects were analyzed, indicating that the percentage of PVP added to the solution influences the diameter size. Figure 3B depicts the diameter dispersion for all configurations associated with the concentration 80/20 (DMAC/PVC), showing the influence of the PVP concentration on the diameter size. Notably, increasing the voltage from 15 to 25 kV led to surface defects, with the appearance of beads attributed to instabilities in the Taylor cone formation.^39^ The water contact angle was analyzed to assess membrane wettability, a critical parameter affecting parameters, such as flux and intermolecular interactions with target contaminants. Figure 3C demonstrates the water contact angle values for all experiments, confirming the hydrophobic nature of electrospun membranes with angles up to 90°.^39^

**Figure 4:**
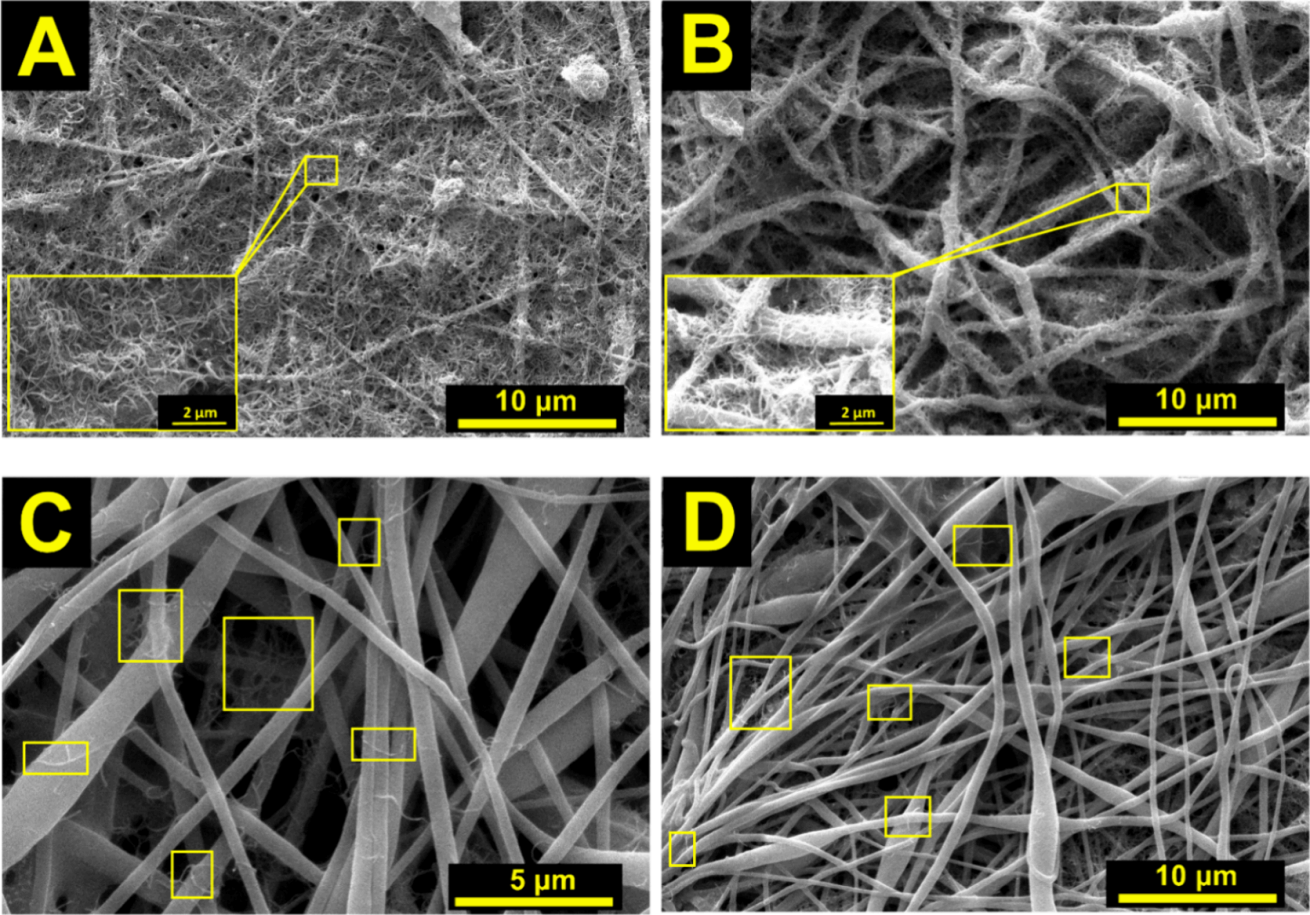
SEM images of PVC/PVP/MWCNT composites with several MWCNTs concentrations: (A) 4.5, (B) 3.6, (C) 2,2, and (D) 1.3 mg/L. Insets highlight the MWCNTs’ presence in the fiber.

Figure 3A presents SEM images illustrating differences related to the concentration of PVP and the applied voltage. Parameters such as fiber diameter and morphological defects were analyzed. The influence of the PVP concentration on fiber diameter is evident from the data in Figure 3B, which displays the diameter dispersion for all configurations associated with the 80/20 (DMAC/PVC) concentration. Meanwhile, given that surface area characteristics are related to fiber diameter, this study finds that the fibers with the best performance exhibit distribution values close to 250 nm. This is comparable to the findings of D. Almeida et al.,^43^ who reported fibers of cellulose acetate modified with cationic surfactant showing diameter values close to 250 nm. Furthermore, their BET analysis shows a surface area close to 11 m^2^/g, a pore size of 7.6 nm, and a pore volume of 1.83 × 10^–3^ cm^3^/g. On the other hand, similar values are expected in our study. It is notable that PVC membranes typically exhibit hydrophobic characteristics.^44,45^ This behavior can be attributed to the distribution of the polymer blend; when lower concentrations of PVP were tested it did not significantly affect the hydrophilicity of the membrane, as indicated by the similar values when the error bar. The composite PVC/PVP/MWCNTs were prepared by spraying the MWCNTs dispersion onto the PVC/PVP membrane with vacuum assistance. Figure 4 provides a comparison of different MWCNT concentrations within the membrane. The quantity of MWCNTs plays a crucial role in their dispersion on the electrospun nanofibers. To analyze the impact of MWCNTs on the membrane morphology, experiments were conducted using four concentrations of MWCNTs over the PVC/PVP membrane.

Figure 4A–D presents SEM images of samples treated with MWCNT dispersions at concentrations of 4.5, 3.6, 2.2, and 1.3 mg/L, corresponding to 100, 80, 50, and 30% relative to the most concentrated dispersion (4.5 mg/L).

Incorporating MWCNTs into the matrix slightly increases the hydrophobicity of the fibers due to their low content. Although MWCNTs typically exhibit high hydrophobic properties, their minimal impact here is attributed to the low filler content on the fibers. A small trend of increasing water contact angle is observed with a higher MWCNT content. The water contact angle for the utilized membrane showed a value of 122.39 ± 5.56°. In contrast, the membranes with MWCNTs exhibited values of 128.25 ± 3.30°, 127.32 ± 4.21°, 124.25 ± 3.28°, and 122.55 ± 5.22° for concentrations of 100, 80, 50, and 30% nanotubes, respectively.

**Figure 5:**
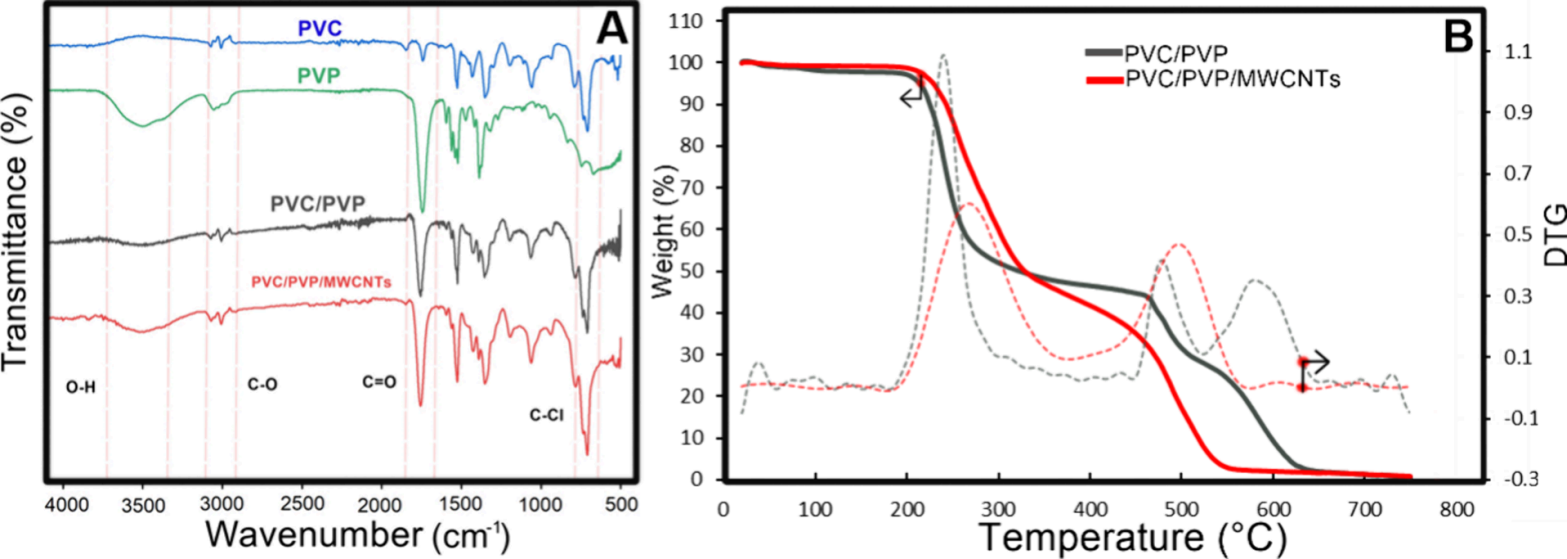
(A) FTIR spectra for PVC, PVP, PVC/PVP membrane, and PVP/PVP/MWCNTs composite. (B) TG and DTG (dashed) curves for PVPC/PVP membrane and PVP/PVC/MWCNTs composite.

We gained insights into the vibrational modes of functional groups within the composite using ATR-FTIR spectroscopy. Each polymer underwent individual analysis and subsequent comparison on a unified chart, as showcased in Figure 5, which presents the infrared spectra of PVC, PVP, PVC/PVP, and PVC/PVP/MCNTs. Notably, the FTIR spectrum highlights distinct peaks corresponding to specific chemical bonds within each polymer. For instance, a peak near 610 cm^–1^, associated with the C–Cl bond, is characteristic of PVC and is observed in both the PVC/PVP blend and PVC/PVP/MWCNT composite. Meanwhile, the vibration mode between 1700 and 1610 cm^–1^, attributed to the C=O (carbonyl group) stretching vibrational mode, is predominantly found in the PVP polymer. However, in the PVC/PVP blend, a shift to a higher frequency indicates significant intermolecular/intramolecular interaction bending, likely resulting from dipole–dipole interactions between C–Cl and C=O bonds. This interaction is evident in the PVC/PVP blend and PVC/PVP/MWCNTs composite. Furthermore, between 2800 and 3000 cm^–1^, a C–H stretching vibrational mode associated with PVC and PVP polymers is observed across all blends. Additionally, between 3300 and 3600 cm^–1^, a group of O–H stretching vibrational modes, characteristic of PVP’s chemical structure, is detected. The thermal properties of PVC/PVP membranes and composite PVC/PVP/MWCNTs were investigated using thermogravimetric analysis (TGA), covering a temperature range from 25 to 750 °C. Figure 5B depicts the thermal stability profiles of both membrane types. Within the temperature range of 200 to 300 °C, an initial degradation stage associated with dehydrochlorination was observed, consistent with previous findings.^30^ Both membranes exhibited similar degradation behavior during this stage, although the PVC/PVP/MWCNT composite demonstrated enhanced stability and a lower weight loss/temperature rate. Additionally, the PVC/PVP/MWCNT membrane exhibited an additional decomposition stage between 450 and 525 °C, maintaining stability up to 750 °C with a residue of 0.69%. In contrast, the PVC/PVP membrane displayed two further decomposition stages, occurring between 450 and 520 °C and starting at 550 °C and extending up to 660 °C, resulting in a higher residue of 39% at 750 °C. The onset of significant weight loss occurred at approximately 321.5 °C for PVC/PVP and 328.4 °C for the PVC/PVP/MWCNTs composite. These findings suggest that incorporating MWCNTs enhances the interaction within the polymeric blend, thereby improving thermal conductivity and promoting a more uniform decomposition process, consequently enhancing thermal stability up to 50% of weight loss.^30^ MWCNTs play a pivotal role in inducing homogeneous decomposition, thus contributing to the observed improvements in thermal stability.

**Figure 6:**
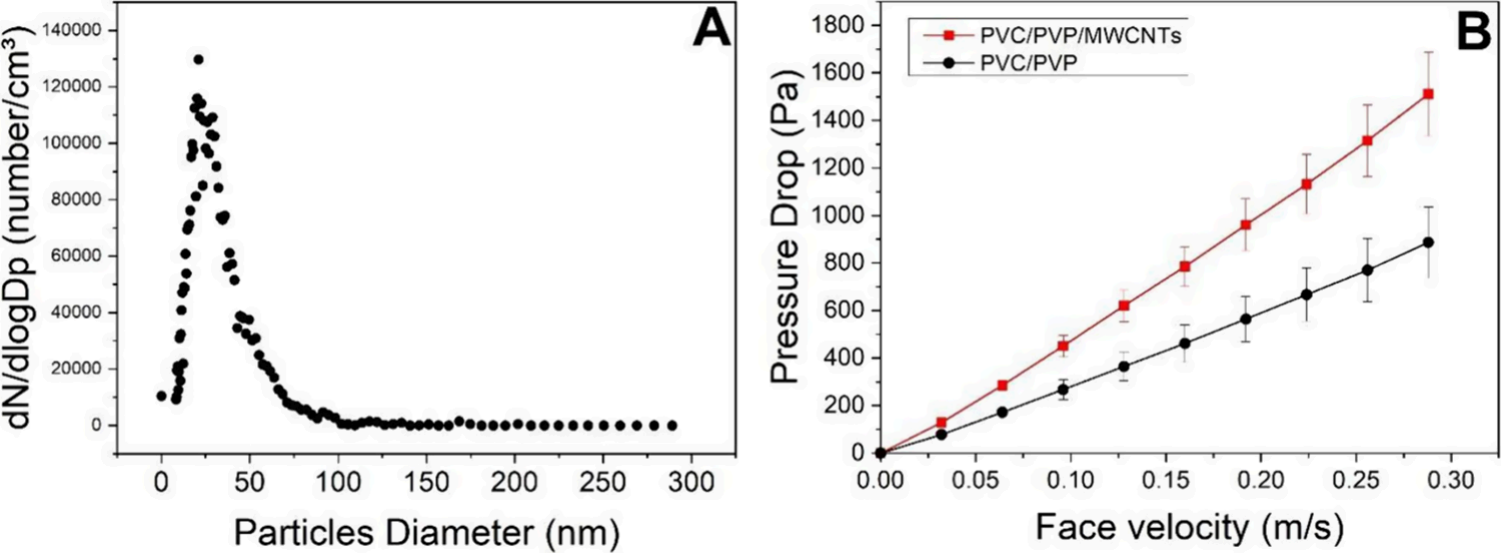
(A) Diameter distribution of NaCl nanoparticles generated by the atomizer and used in the air filter test. (B) Average pressure drop as a function of face velocity using PVC/PVP and PVC/PVP/MWCNTs.

To assess the filtration efficiency for collecting nanoparticles, sodium chloride (NaCl) nanoparticles were generated by using an atomizer. The size distribution of these nanoparticles is presented in Figure 6A, with an average diameter of approximately 45 nm, and the results of permeability tests are thoughtfully illustrated in Figure 6B. The data summary can be accessed in Table 1, which shows that a resume of the principal filtration properties found for the membranes developed. Permeability is an essential parameter in the filtering process to indicate the facility to transmit fluids in a filter. An increase in the pressure drop was expected due to the larger thickness. In this case, a slight difference of close to 60 μm on each kind of membrane exists. However, an increase in the pressure drop was associated with the incorporation of MWCNTs. The SEM images in Figure 4B show a good dispersion of MWCNTs.

**Table 1:** Thickness (μm), Pressure Drop (Δ*P*/*L*), Permeability Constant (k), and Global Collection Efficiency (%) (Particle Diameter 7 to 300 nm) for PVC/PVP Nanofibers Obtained by Electrospinning with and without Carbon Nanotubes

measurement (mean)	PVP/PVP	PVC/PVP/MWCNTs
thickness (μm)	160.25 ± 22.30	202.47 ± 11.02
Δ*P* (Pa)	532.00 ± 45.58	758.73 ± 123.02
*K* × 10^–12^ m^2^	4.25 ± 2.99	2.11 ± 0.26
total efficiency (%)	96.30 ± 2.60	96.99 ± 0.67
QF	0.0061	0.0046

The constant of permeability shows that the PVC electrospun membrane presents higher permeability than PVC/PVP/MWCNTs. Additionally, the constant permeability (*k*) found is of the same order as HEPA (high-efficiency particulate air) 10–11 m^2^ and ULPA (ultra low penetration air) filters 10^–12^ m^2^.^40^ D. Almeida et al.^41^ produced nanofibers by electrospinning composed of cellulose acetate and cationic surfactant and tested in the same system; they obtained a “*k*” coefficient of 10^–11^ m^2^ at 1.6 cm/s.

The membranes containing MWCNTs experience pressure drops significantly higher than those of their nanotube-free counterparts. This behavior aligns with expectations because the nanotubes occupy empty spaces within the membrane, reducing its porosity and limiting its permeability. Incorporating MWCNTs creates a physical barrier that fills a substantial portion of the gaps between fibers. This approach shows a notable dispersion of MWCNTs onto the fibers, allowing precise control to reach the desired saturation level. Such precision is essential for filtration processes and potential uses in adsorbing volatile organic compounds. Leveraging the extensive surface area of MWCNTs, we expect a promising synergy between the matrix and MWCNTs, ensuring an effective performance in these processes. In a study conducted by Bortolassi and colleagues,^41^ three commercial filters meeting HEPA standards were comprehensively compared. This comparison involved permeability testing at various velocities ranging from 1 to 16 cm/s. The results showed that the pressure drop reached 269 ± 16, 397 ± 12, and 418 ± 11 Pa at a velocity of 5 cm/s for the commercial filters.

In contrast, the pressure drops for PVC/PVP and PVC/PVP/MWCNT membranes were approximately 150 and 200 Pa, indicating significantly improved permeability at these velocities, which are relatively high within the scope of these studies. The permeability constant found is higher than that found in glass fiber HEPA and ULPA filters, which are about 10^–11^.^46^ Hence, the operation life of the PVC/PVP membranes should be about 6 months to 1 year, like HEPA filters composed of glass fiber and borosilicate microfibers, which present similar permeability. In a study by Liu Chog and collaborators,^47^ a nanofiber membrane was created from polyacrylonitrile (PAN) and compared to two commercial filters. At a velocity of 0.21 m/s, the PAN electrospun nanofibers exhibited pressure drops of 133 and 206 Pa. In contrast, the commercial filter showed substantially higher pressure drops, reaching 299 and 809 Pa values.

**Figure 7:**
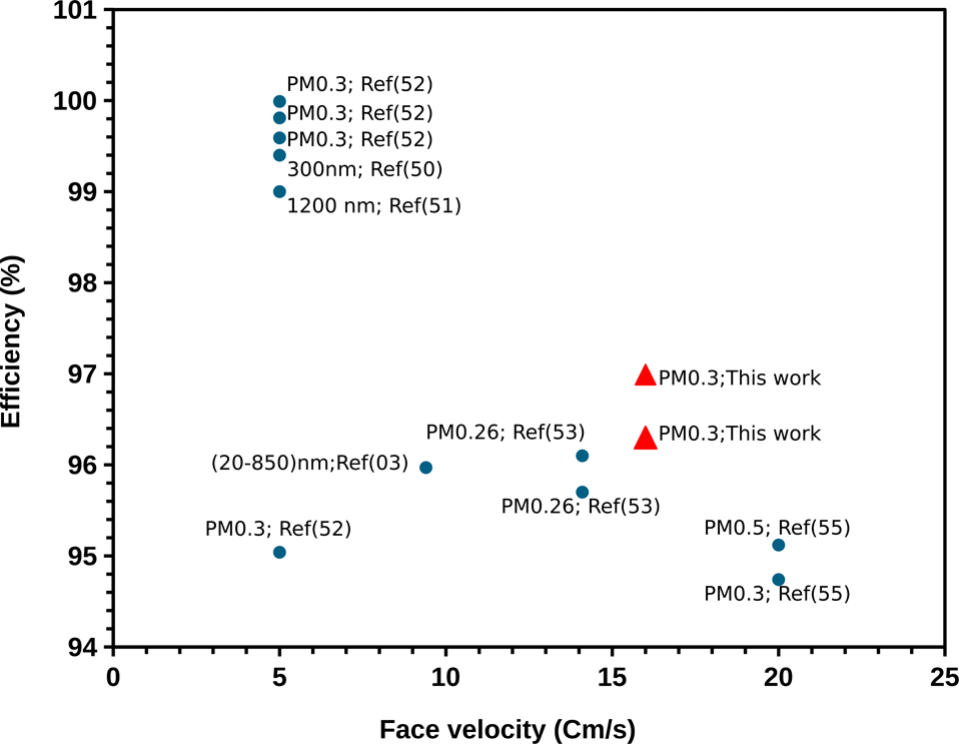
Chart contrasts several relevant studies,^3,48−52^ showing that most focus on face velocities around 5 cm/s, achieving satisfactory efficiency results. However, it highlights some articles, including our own, that maintain high efficiency, even at higher velocities.

On the other hand, in Figure 7, it can be seen that many studies focus on evaluating filters at low face velocities. However, this image highlights that even with increased velocities, a considerably high efficiency is maintained. This suggests that increasing the velocity would minimally impact efficiency loss. This finding is particularly attractive for indoor contaminant filtration systems, as it would allow for maintaining contaminant-free spaces for extended periods.

Importantly, when assessing efficiency in capturing PM2.5 particles, the electrospun nanofibers demonstrated an impressive efficiency rate of 96.99%. In contrast, the commercial filters achieved values ranging from 16.93 to 99.58%. This analysis highlights the significantly superior permeability of electrospun fibers compared to conventional commercial filters.

## Conclusions

The concentration 80/20 (DMAC/PVC) performed better in smaller diameters and morphological defects, while the concentration 85/15 showed an enormous concentration of beads. The formulation 80/20 DMAC/PVC plus 3% wt of PVP and 15 kV of voltage applied (80/20pvc3pvp1) shows the most exciting results regarding morphology, small diameter, and fewer defects. Infrared spectroscopy analysis proved the presence of the polymers in the membrane and simultaneously revealed the interaction between the C–Cl groups of PVC and the C=O groups of PVP. The excellent interaction between the MWCNTs and the PVC/PVP electrospun fibers was also demonstrated. Considering the method used in the deposition, it can be understood that the nanotubes have excellent electrical adhesion to the electrospun fibers, remaining in the polymer matrix rather than in DI water. The filtration performance tests showed that a possible application of this nanofiber could be considered for trapping particles smaller than 300 nm. Good filtration performance for NaCl particles was tested, and an efficiency of about 96% was obtained at a pressure drop of 780 Pa and a flow rate of 0.16 m/s with the MWCNT-containing samples. The calculated permeability is similar to that obtained using the same technique and is 10^–12^ m^2^. This research provides information for future designs of filter media materials once the PVC/PVP/MWCNT nanofibers can be used as filter media for various indoor various applications.

## Supporting Information Available

Experimental design considered 3 parameters: concentration DMAC/PVC with 3 levels and digital microscope images of all the samples analyzed in the experimental design (PDF)
