# Supplementary material for: Enhanced Air Filtration Efficiency through Electrospun PVC/PVP/MWCNTs Nanofibers: Design, Optimization, and Performance Evaluation
Source: ACS Omega. 2024 Aug 24;9(36):37771–9. doi: 10.1021/acsomega.4c03628 (PMC11391459; doi:10.1021/acsomega.4c03628)
Supplement: Supplementary file 1 — ao4c03628_si_001.pdf [file ao4c03628_si_001.pdf]

Electronic Supplementary Information

Enhanced Air Filtration Efficiency through Electrospun PVC/PVP/MWCNTs Nanofibers: Design, Optimization, and Performance Evaluation

Armando A. Escriba Flores[1], Daniela Sanches de Almeida[2], Monica Lopes Aguiar[2], Carlos Eduardo Cava[1*]

[1]Federal University of Technology – Paraná. Av. Dos Pioneiros, 3131, Londrina - PR, 86036-370, Brazil.

[2] Federal University of São Carlos, Rod. Washington Luiz, km 235, SP310, São Carlos - SP, 13565-905, Brazil.

*Corresponding author – carloscava@utfpr.edu.br

Table S1. The experimental design parameters, the experimental design considered 3 parameters: concentration DMAC/PVC with 3 levels.

Experime nt	A	B	C	DMAC/PVC	PVP %Wt	Voltage (kW)	Code
1	-1	-1	-1	75/25	3	15	75/25PVC3PVP 15
2	0	-1	-1	80/20	3	15	80/20PVC3PVP 15
3	1	-1	-1	85/15	3	15	85/15PVC3PVP 15
4	-1	0	-1	75/25	5	15	75/25PVC5PVP 15
5	0	0	-1	80/20	5	15	80/20PVC5PVP 15
6	1	0	-1	85/15	5	15	85/15PVC5PVP 15
7	-1	1	-1	75/25	7	15	75/25PVC7PVP 15
8	0	1	-1	80/20	7	15	80/20PVC7PVP 15
9	1	1	-1	85/15	7	15	85/15PVC7PVP 15
10	-1	-1	1	75/25	3	25	75/25PVC3PVP 25
11	0	-1	1	80/20	3	25	80/20PVC3PVP 25
12	1	-1	1	85/15	3	25	85/15PVC3PVP 25
13	-1	0	1	75/25	5	25	75/25PVC5PVP 25
14	0	0	1	80/20	5	25	80/20PVC5PVP 25
15	1	0	1	85/15	5	25	85/15PVC5PVP 25
16	-1	1	1	75/25	7	25	75/25PVC7PVP

							25
17	0	1	1	80/20	7	25	80/20PVC7PVP 25
18	1	1	1	85/15	7	25	85/15PVC7PVP 25

Figure S1. Digital microscope images of all the samples analyzed in the experimental design.

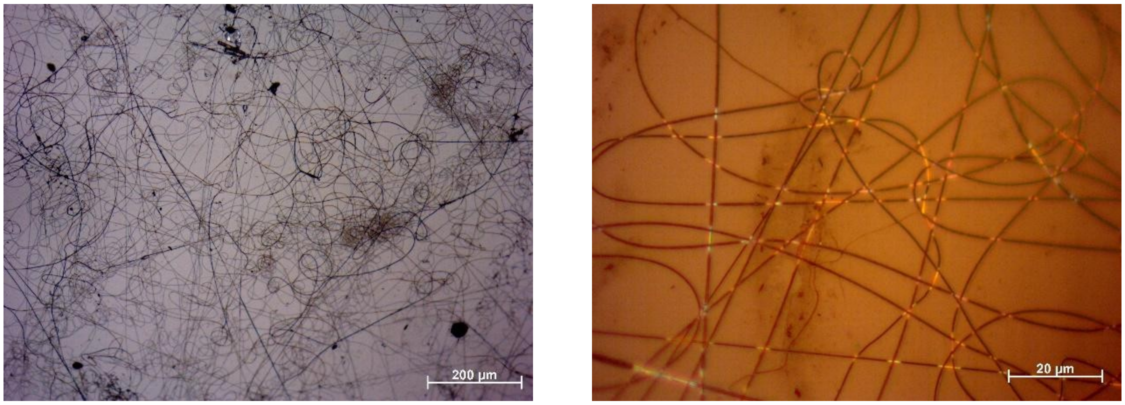

(a) Experiment 2 80/20PVC3PVP15

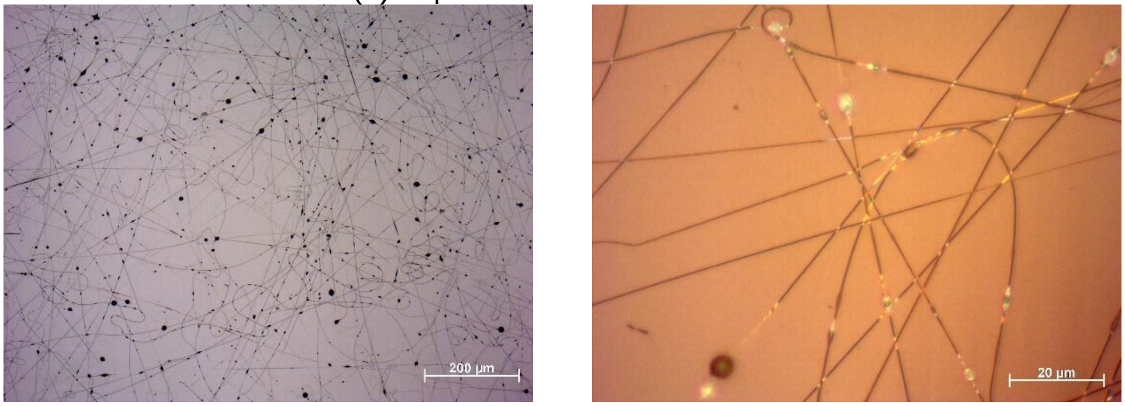

(b) Experiment 3 85/15PVC3PVP15

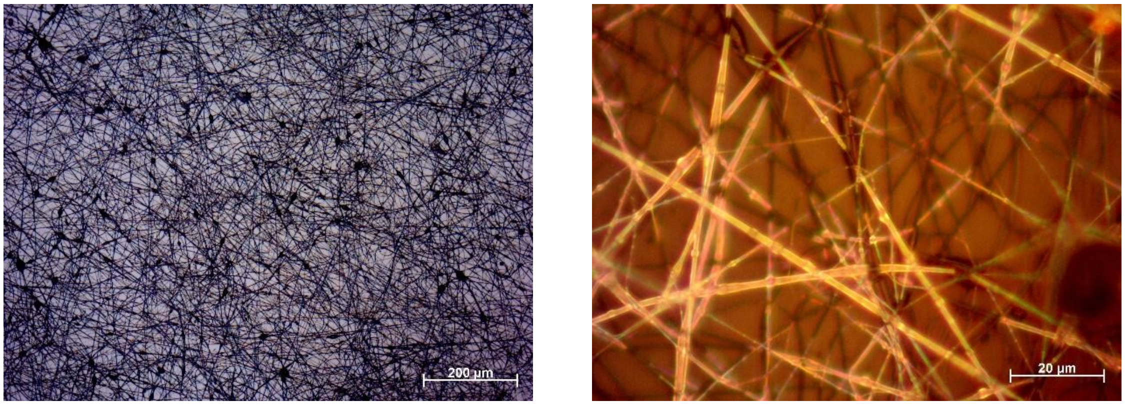

(c) Experiment 5 80/20PVC5PVP15

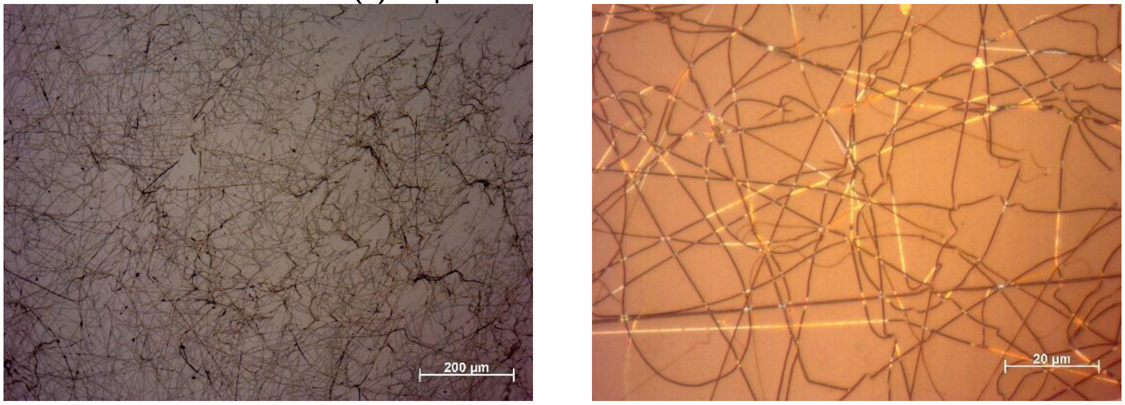

(d) Experiment 6 85/15PVC5PVP15

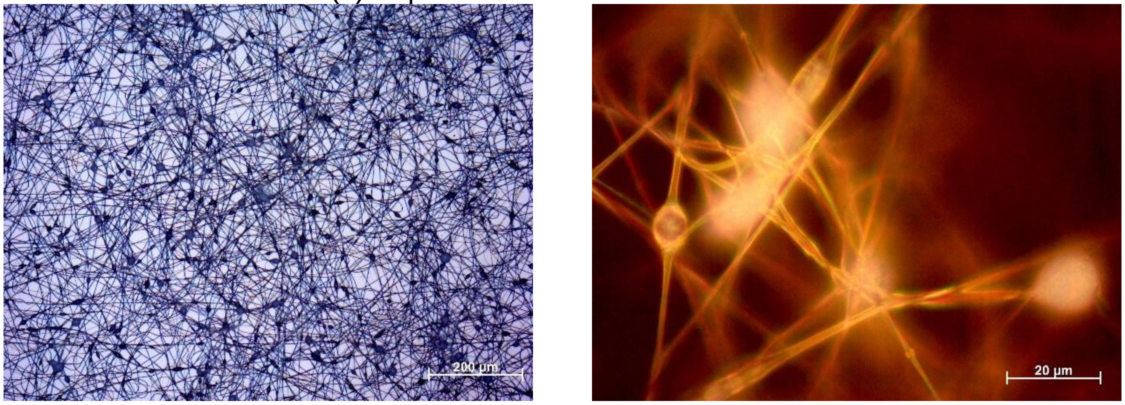

(e) Experiment 8 80/20PVC7PVP15

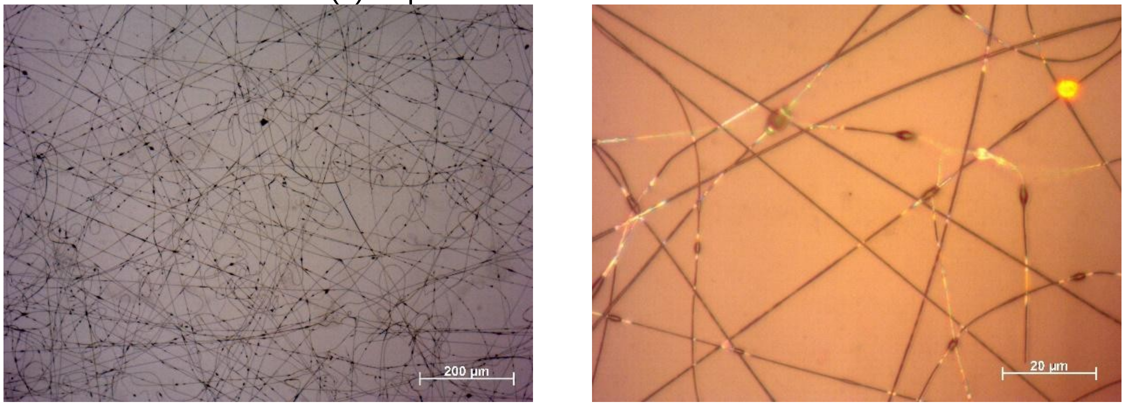

(f) Experiment 9 85/15PVC7PVP15

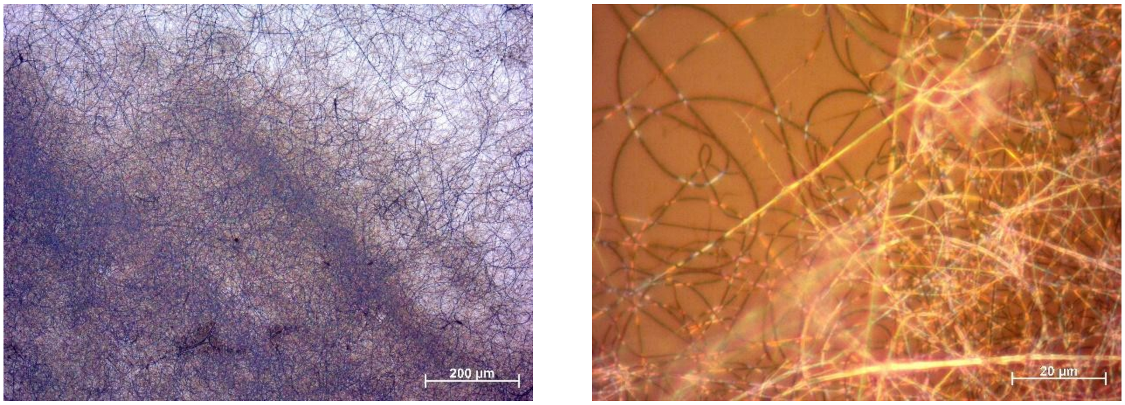

(g) Experiment 11 80/20PVC3PVP25

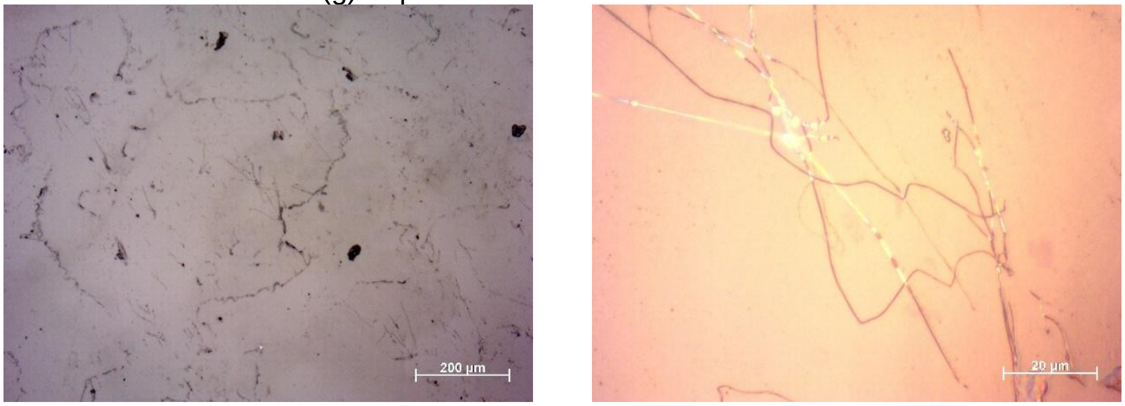

(h) Experiment 12 85/15PVC3PVP25

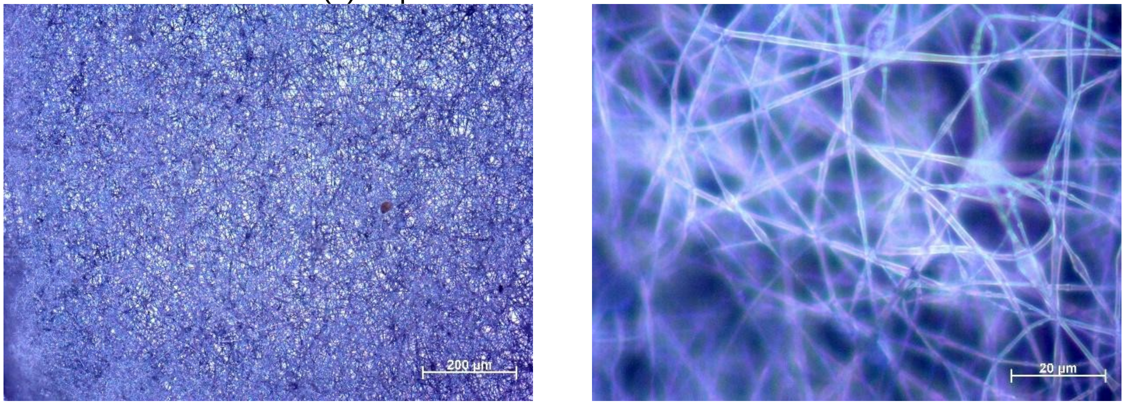

(i) Experiment 14 80/20PVC5PVP25

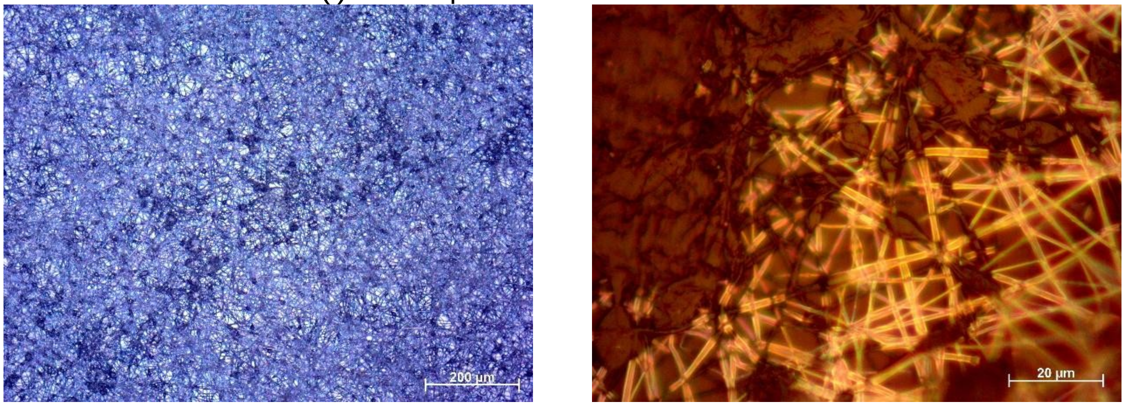

(j) Experiment 17 80/20PVC7PVP25

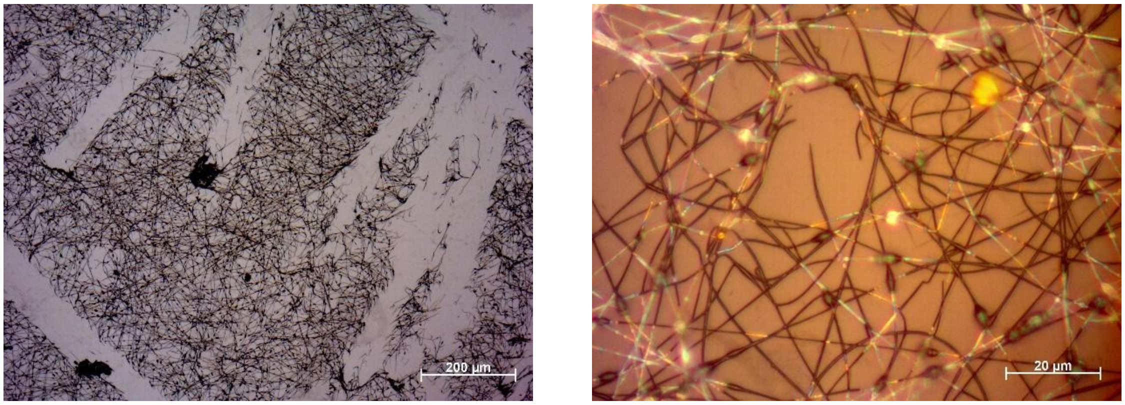

(k) Experiment 18 85/15PVC7PVP25

All the experiments with the concentration 75/25 dint allow the formation of electrospun fibers. the viscosity of the sample was too high to allow it.

The thermal analysis was conducted by thermogravimetric, where it was possible to analyze both membrane PVC/PVP and PVC/PVP/MWCNTs from 25°C up to 750°C. Figure 3(B) shows the thermal stability of PVC/PVC membranes and composite PVC/PVP/MWCNTs. Near 200°C up to 300°C is evidenced as the first stage of degradation associated with the dehydrochlorination phenomenon [55].Both membranes presented similar behavior in this stage of decomposition. However, PVC/PVP/MWCNTs evidenced better stability and the weight loss/temperature rate. PVC/PVP/MWCNTs membrane presented one more stage of decomposition, from 450°C to 525°C, and remained stable until 750°C with a residue of 0.69%. PVC/PVP evidenced two more decompositions at 450 to 520°C, starting at 550°C, and it is prolonged up to reach 660°C. The residue at 750°C in PVC/PVP was estimated at 0.39%. The samples presented 50% weight loss in 321.5°C for the PVC/PVP membrane and 328.4°C for the composite PVC/PVP/MWCNTs. The influence of the MWCNTs shows better interaction between the polymeric blend, improving the thermal conductivity and inducing a homogeneous decomposition, leading to better thermal stability up to 50% weight loss.
